# Retraction: Insulin-induced Drosophila S6 kinase activation requires phosphoinositide 3-kinase and protein kinase B

**DOI:** 10.1042/BJ20030577_RET

**Published:** 2025-04-01

**Keywords:** *Drosophila*, insulin signalling, phosphatase and tensin homologue deleted on chromosome 10 (PTEN), phosphoinositide 3-kinase (PI3K), protein kinase B (PKB), S6 kinase (S6K)

The authors would like to retract their article “Insulin-induced Drosophila S6 kinase activation requires phosphoinositide 3-kinase and protein kinase B” (DOI: 10.1042/bj20030577)” from the *Biochemical Journal*, having been alerted by a reader of similarities in Western blot images between several figures. Given the 20 + years that have passed since the work producing these data was carried out in Professor Alessi’s laboratory at the University of Dundee, the corresponding author and the research integrity group in the School of Life Sciences at the University of Dundee have not been able to obtain the original data for this article, so feel that the best course of action given the concerns is to retract the paper. The Publisher agrees with the retraction. The authors sincerely apologise for any inconvenience caused.

The similarities identified are:

- Figure 1B dPKB appears as part of Figure 7A dS6K.
- Figure 2B dS6K appears as Figure 2B dPKB with horizontal transformation.
- Figure 4A Kc167 cells dS6K appear in part as S2 cells dPKB in the same Figure with horizontal transformation.
- Figure 4B Kc167 cells dS6K appear as S2 cells dS6K in the same Figure with horizontal transformation.
- Figure 7A Kc167 cells p-dPKB appear in part as Figure 7B S2 cells p-dPKB with horizontal transformation.

The authors would like to highlight that the main conclusions reported in this article remain valid. The findings that the activation of dS6K in response to insulin requires dPKB have been thoroughly validated by subsequent research in the Drosophila S2 cell model [Miron et al., 2003 (DOI: 10.1128/MCB.23.24.9117–9126.2003), Potter et al., 2003 (DOI: 10.1042/bst0310584) and Yang et al., 2006 (DOI: 10.1073/pnas.0602282103)] and in mammalian cells by numerous laboratories [reviewed in Panwar et al., 2023 (DOI: 10.1038/s41392-023-01608-z)].

